# Collectives of diagnostic biomarkers identify high-risk subpopulations of hematuria patients: exploiting heterogeneity in large-scale biomarker data

**DOI:** 10.1186/1741-7015-11-12

**Published:** 2013-01-17

**Authors:** Frank Emmert-Streib, Funso Abogunrin, Ricardo de Matos Simoes, Brian Duggan, Mark W Ruddock, Cherith N Reid, Owen Roddy, Lisa White, Hugh F O'Kane, Declan O'Rourke, Neil H Anderson, Thiagarajan Nambirajan, Kate E Williamson

**Affiliations:** 1 Centre for Cancer Research & Cell Biology, Queens University Belfast, 97 Lisburn Road, Belfast, BT9 7BL, Northern Ireland; 2 Department of Urology, Belfast City Hospital, 75 Lisburn Road, Belfast, BT9 7AB, Northern Ireland; 3 Molecular Biology, Randox Laboratories Ltd, Diamond Road, Crumlin, BT29 4QY, Northern Ireland; 4 Department of Pathology, Belfast City Hospital, 75 Lisburn Road, Belfast, BT9 7AB, Northern Ireland; 5 Department of Pathology, Royal Victoria Hospital, 274 Grosvenor Road, Belfast, BT12 6AB, Northern Ireland

**Keywords:** hematuria, biomarker, risk stratification, Random Forests Classifier, hierarchical clustering, feature selection, urothelial cancer, proteinuria

## Abstract

**Background:**

Ineffective risk stratification can delay diagnosis of serious disease in patients with hematuria. We applied a systems biology approach to analyze clinical, demographic and biomarker measurements (n = 29) collected from 157 hematuric patients: 80 urothelial cancer (UC) and 77 controls with confounding pathologies.

**Methods:**

On the basis of biomarkers, we conducted agglomerative hierarchical clustering to identify patient and biomarker clusters. We then explored the relationship between the patient clusters and clinical characteristics using Chi-square analyses. We determined classification errors and areas under the receiver operating curve of Random Forest Classifiers (RFC) for patient subpopulations using the biomarker clusters to reduce the dimensionality of the data.

**Results:**

Agglomerative clustering identified five patient clusters and seven biomarker clusters. Final diagnoses categories were non-randomly distributed across the five patient clusters. In addition, two of the patient clusters were enriched with patients with 'low cancer-risk' characteristics. The biomarkers which contributed to the diagnostic classifiers for these two patient clusters were similar. In contrast, three of the patient clusters were significantly enriched with patients harboring 'high cancer-risk" characteristics including proteinuria, aggressive pathological stage and grade, and malignant cytology. Patients in these three clusters included controls, that is, patients with other serious disease and patients with cancers other than UC. Biomarkers which contributed to the diagnostic classifiers for the largest 'high cancer- risk' cluster were different than those contributing to the classifiers for the 'low cancer-risk' clusters. Biomarkers which contributed to subpopulations that were split according to smoking status, gender and medication were different.

**Conclusions:**

The systems biology approach applied in this study allowed the hematuric patients to cluster naturally on the basis of the heterogeneity within their biomarker data, into five distinct risk subpopulations. Our findings highlight an approach with the promise to unlock the potential of biomarkers. This will be especially valuable in the field of diagnostic bladder cancer where biomarkers are urgently required. Clinicians could interpret risk classification scores in the context of clinical parameters at the time of triage. This could reduce cystoscopies and enable priority diagnosis of aggressive diseases, leading to improved patient outcomes at reduced costs.

## Background

The number of patients presenting with hematuria is progressively increasing in our aging population and the diagnosis of serious diseases in some of these patients can be delayed when triage is ineffective [1]. Therefore, novel alternative risk stratification approaches are needed [2].

Hematuria, that is, the presence of blood in urine, is a presenting symptom for a variety of diseases. The final diagnosis for hematuric patients ranges from no diagnosis, through benign conditions including urinary infection, stone disease, benign prostate enlargement (BPE) to renal diseases and malignant causes. Urothelial cancer (UC), the most common malignancy in hematuric patients, is the fourth most common cancer in men and was the estimated cause of death in 150,200 people worldwide in 2008 [3]. Bladder cancer is associated with many risk factors [2]. Smoking increases the risk of UC fourfold and cessation of smoking is associated with a decreased risk [2].

The risk parameters that are currently used to tailor follow-up for patients diagnosed with UC, include pathological parameters, that is, grade, stage and associated carcinoma *in situ *(CIS), together with resistance to Bacille Calmette-Guerin treatment. At the time of diagnosis, approximately 70% of patients diagnosed with UC have tumors that are pathologically staged as pTa, pT1 or CIS, that is, non-muscle invasive (NMI) disease. The remaining patients present with muscle invasive UC (MI UC) which has a high-risk of progression to a more life threatening disease [2,4]. Unfortunately, it is not always possible to predict correctly the outcome for patients. This is largely attributable to the molecular heterogeneity within tumors which means that a spectrum of outcomes, spanning from negligible risk to life threatening prognosis, exist within the same pathological classification. For this reason, all patients with NMI disease have frequent surveillance cystoscopies and those with MI UC have radiological surveillance for lymph node recurrence or distant metastasis [2].

Cystoscopy is the gold standard for the detection and surveillance of NMI UC [2]. However, this procedure is costly and invasive for the patient. Further, it requires a significant clinical input and has its own shortcomings [2,5]. Cytology, another diagnostic test for bladder cancer, detects the presence of malignant cells in urine. Although cytology has high specificity, it has insufficient sensitivity to stand alone as a diagnostic test for UC in patients presenting with hematuria [2]. Three diagnostic bladder cancer biomarkers, Nuclear Matrix Protein 22 [6], Bladder Tumor Antigen (BTA) [7] and Fibrinogen Degradation Product [8] have Food and Drug Administration (FDA) approval. However, these biomarkers are not in use in routine practice as diagnostic biomarkers for UC because of their limited specificity. There is, therefore, a strong clinical need for urine-based tests which can at least risk stratify and, if possible, be diagnostic in hematuric patients [2].

Researchers often combine multiple tests, genes or biomarkers [9-11]. However, it is not possible to predict intuitively how multiple measurements will collectively reflect the underlying biological heterogeneity in complex diseases, such as UC. Complex diseases consist of multiple components which interact to produce emergent properties that the individual components do not possess. The difficulties to date with large amounts of patient biomarker data are that they do not manage or group all patients in a clinically meaningful way. Systems biology is based on the assumption that interactions among molecular components need to be integrated in order to obtain a functional understanding of physiological properties [12,13]. In this paper we used a systems approach, that is, clustering and Random Forests Classification (RFC), to analyze a comprehensive dataset collected from 157 hematuric patients: 80 patients with UC and 77 controls with a range of confounding pathologies.

When we allowed the patients to cluster naturally on the basis of their individual biomarker profiles this resulted in five patient clusters with a non-random distribution of risk characteristics. Three of these patient clusters were enriched with patients with cancer-risk characteristics. The remaining two patient clusters were enriched with patients with non-cancer characteristics.

## Methods

### Patient information and samples

We analyzed data collected during a case-control study approved by the Office for Research Ethics Committees Northern Ireland (ORECNI 80/04) and reviewed by hospital review boards. The study was conducted according to the Standards for Reporting of Diagnostic Accuracy (STARD) guidelines [14,15]. Written consent was obtained from patients with hematuria who had recently undergone cystoscopy or for whom cystoscopy was planned. Patients (n = 181) were recruited between November 2006 and October 2008 [9]. A single consultant pathologist undertook a pathological review of the diagnostic slides for all bladder cancer patients. The following patients were excluded from our analyses: 19 patients with a history of bladder cancer who were disease-free when sampled; one patient who had adenocarcinoma; one patient who had squamous cell carcinoma; and three patients ≥ 85 years old. We, therefore, analyzed data from 157 patients. A single consultant cytopathologist reviewed the cytology from 74 bladder cancer and 65 control patients. There were insufficient cells for diagnosis in 18/157 patients.

The final diagnosis for each of the 157 patients was based on history, physical examination, urinary tract radiological and endoscopy findings and the pathological reports relating to biopsy or resection specimens. For 36/157 (23%) patients, it was not possible to identify the underlying cause for hematuria, even after detailed investigations, including cystoscopy and radiological imaging of the upper urinary tract. These patients were assigned to the 'no diagnosis' category. The remaining patients were assigned into one of the following six categories: 'benign pathologies', 'stones/inflammation', 'BPE', 'other cancers', 'NMI UC' or 'MI UC'. For analyses purposes, we grouped 'no diagnosis', 'benign pathologies', 'stones/inflammation' and 'BPE' together as non-life threatening diagnoses, and grouped 'other cancers', 'NMI UC' and 'MI UC' as life threatening diagnoses (Table 1).

**Table 1:** Final diagnosis categories.

Individual patient final diagnosis	n	Final diagnosis category	Group	Total
No diagnosis	36	No diagnosis	NLT	36
Category total				36

Fistula	1	Benign	NLT	
Endometriosis	1	Benign	NLT	
Trauma	1	Benign	NLT	
Renal trauma	1	Benign	NLT	
Renal cyst	1	Benign	NLT	
Squamous Metaplasia	1	Benign	NLT	
Category total				6

Stone	9	Stones/inflammation	NLT	
Stone(s) with inflammation	2	Stones/inflammation	NLT	
Stone with UTI	1	Stones/inflammation	NLT	
Urinary tract infection	1	Stones/inflammation	NLT	
Inflammation	4	Stones/inflammation	NLT	
Category total				17

BPE with stone	2	BPE	NLT	
BPE	10	BPE	NLT	
Category total			NLT	12

Renal cell carcinoma with BPE	1	Other cancers	LT	
Renal cell carcinoma	2	Other cancers	LT	
Prostate cancer	3	Other cancers	LT	
Category total				6

UC kidney ureter	1	NMI UC	LT	
NMI TCC with stone	2	NMI UC	LT	
NMI TCC with BPE	1	NMI UC	LT	
NMI TCC	58	NMI UC	LT	
Category total				62

MI UC with stone	2	MI UC	LT	
MI UC with BPE	2	MI UC	LT	
MI UC	14	MI UC	LT	
Category total				18

TOTAL				157

The final diagnosis was determined individually for each patient. The decision was based on history, physical examination, urinary tract radiological and endoscopy findings and also the pathological reports relating to biopsy or resection specimens. Based on their final diagnosis, each patient was assigned to one of the seven final diagnosis categories. For statistical purposes these categories were split into non-life threatening (NLT) or life threatening (LT) groups. BPE, benign prostate enlargement; MI muscle invasive; n, number; NMI, non-muscle invasive; TCC transitional cell carcinoma; UC, urothelial cancer; UTI, urinary tract infection.

## Biomarker measurement

Biomarker measurements were undertaken on anonymized samples at Randox Laboratories Ltd. For each patient, we measured 29 biomarkers; 26 were measured in triplicate (Table 2). Samples were stored at -80^°^C for a maximum of 12 months prior to analysis. Creatinine levels (µmol/L) were measured using a Daytona RX Series Clinical Analyzer (Randox) and Osmolarity (mOsm) was measured using a Löser Micro-Osmometer (Type 15) (Löser Messtechnik, Germany). Total protein levels (mg/ml) in urine were determined by the Bradford assay A_595nm _(Hitachi U2800 spectrophotometer) using bovine serum albumin as the standard. We classified proteinuria as total urinary protein >0.25mg/ml [16]. Eighteen biomarkers in urine, and carcino-embryonic antigen (CEA) and free prostate specific antigen (FPSA) in serum were measured using Randox Biochip Array Technology (Randox Evidence © and Investigator ©), which are multiplex systems for protein analysis [17]. An additional four biomarkers were measured using commercially available ELISAs. Epidermal growth factor (EGF) and the matrix metalloproteinase 9 neutrophil-associated gelatinase lipocalin (MMP9-NGAL) complex were measured using in-house ELISAs (Table 2).

**Table 2:** Biomarkers.

Biomarker	Units	Analysis	Clinical application
Protein	mg/ml	Bradford Assay	Kidney disease
Creatinine	µmol/L	Daytona RX Series Clinical Analyzer (Randox)	Kidney disease
Osmolality	mOsm	Löser Micro-Osmometer (Type 15) (Löser Messtechnik, Germany)	Kidney disease
Bladder tumor antigen (BTA)	U/ml	ELISA (Polymedco)	UC diagnosis
Carcino-embryonic antigen (CEA) (serum)	ng/ml	BAT	Monitoring colorectal cancer
_‡_Cytokeratin 18 (CK18)	ng/ml	ELISA (USCNLIFE Science & Technology Co. Ltd)	N/A
C-reactive protein (CRP)	ng/ml	BAT	Acute inflammation/infection
D-dimer	ng/ml	BAT	Pulmonary embolus
Epidermal growth factor (EGF)	pg/ml	ELISA (in house)	UC prognosis
_‡_FAS	pg/ml	ELISA (Raybio, Inc)	N/A
_‡_Hyaluronidase (HA)	ng/ml	ELISA (Echelon Biosciences Inc)	N/A
Interleukin-1α (IL-1 α)	pg/ml	BAT	N/A
Interleukin-1β (IL-1 β)	pg/ml	BAT	N/A
Interleukin-2 (IL-2)	pg/ml	BAT	N/A
Interleukin-4 (IL-4)	pg/ml	BAT	N/A
Interleukin-6 (IL-6)	pg/m	BAT	N/A
InterleukinL-8 (IL-8)	pg/ml	BAT	N/A
Monocyte chemoattractant protein-1 (MCP-1)	pg/ml	BAT	N/A
Matrix metalloproteinase 9 (MMP9)	ng/ml	BAT	N/A
MMP-9NGAL complex	N/A	ELISA (in house)	N/A
Neutrophil-associated gelatinase lipocalin (NGAL)	ng/ml	BAT	Kidney disease
Neuron specific enolase (NSE)	ng/ml	BAT	
Free prostate specific antigen (FPSA) (serum)	ng/ml	BAT	Prostate cancer
Thrombomodulin (TM)	ng/ml	BAT	N/A
Tumor necrosis factor α (TNFα)	pg/ml	BAT	N/A
Soluble tumor necrosis factor receptor 1 (sTNFR1)	ng/ml	BAT	N/A
Soluble tumor necrosis factor receptor 2 (sTNFR2)	ng/ml	BAT	N/A
Vascular endothelial growth factor (VEGF)	pg/ml	BAT	angiogenesis
Von Willeband factor (vWF)	IU/ml	BAT	N/A

Biomarkers were measured in triplicate except for those marked ‡ for which only a single analysis was undertaken. Twenty of the 29 biomarkers were measured using Biochip Array Technology (BAT) which facilitates the simultaneous analyses of multiple proteins [17]. ELISA, enzyme-linked immunosorbent assay; UC urothelial cancer.

### Data representation

Data were represented by a matrix *X *with 157 rows and 29 columns, for example, *X*(3,5) contained the measurement for patient number 3 and biomarker number 5. In order to simplify the notation, we denoted by *X*(j,) the 29 dimensional feature vector for patient *j *and by *X*(,*k*) the 157 dimensional feature vector for biomarker *k*.

### Identification of patient clusters

Patients were separated into clusters according to the similarities of their 29 biomarkers using a hierarchical clustering with a Canberra distance and a Mcquitty clustering [18]. Therefore, each patient's profile vector was derived from the levels of the 29 biomarkers in their samples, for example, *X*(*i*,) as a profile vector for patient *i*. To demonstrate the robustness of the observed clusters, we repeated the same analysis 100 times using only a bootstrap subset of the patients to conduct the clustering.

### Chi-square tests

We explored the distribution of final diagnoses and known cancer risk characteristics across the patient clusters. We then constructed five cross-tables in which the patient clusters were listed in rows; and the final diagnosis category, absence/presence of proteinuria, pathological stage, pathological grade, or absence/presence of malignant cytology, was listed in columns. When the number of observed counts was <5 in >80% of cells in any of these tables, we merged groups as previously described (Table 1), prior to undertaking Chi-square analysis.

### Identification of biomarker clusters

To allow us to exploit the full complement of biomarker data for subsequent classifications, we conducted hierarchical clustering to identify substructures within the 29 biomarkers themselves. That means for each biomarker *k *we used *X*(,*k*) as a profile vector to conduct an agglomerative clustering for the 29 biomarkers. Thus each biomarker's profile vector was based on the levels of the biomarker measured in each of the 157 patients. On the assumption that biomarkers within individual biomarker clusters would be similar to each other and, hence, contain redundant biological information about patients, we subsequently used one biomarker from each cluster for the classification of individual patient clusters and patient subpopulations, as described in the next section.

### Random forest classification (RFC)

As our classification method, we used RFC which is an ensemble method consisting of multiple decision trees which, taken together, can be used to assign each patient into either of two categories. The overall classification of the RFC is obtained by combining the individual votes (classifications) of all individual trees, that is, by a majority vote [19,20]. We used the biomarker clusters to estimate the effective dimension of a feature set for the classification of the patient subpopulations. Each RFC was, therefore, constructed using one biomarker from each of the seven biomarker clusters. We estimated the area under the receiver operating characteristic curve (AUROC) by using out-of-bag samples, which means that the trees of a RFC were trained with bootstrap data which omit approximately one-third of the cases each time a tree is trained. These samples, called out-of-bag samples, are used as test data sets to estimate the classification errors [19].

As a benchmark, we first determined the classification error and the AUROC of RFCs with 1,000 trees for all possible collectives of biomarkers for the total population, that is, 157 patients. Second, we determined classification errors and AUROCs for RFCs for each of the three largest natural patient clusters. Third, we determined classification errors and AUROCs of RFCs for 14 clinically defined subpopulations of patients.

We assumed that clusters/subpopulations with similar contributory biomarkers to their classifiers were more homogeneous than subpopulations with different contributory biomarkers. On this basis, we compared contributory biomarkers to the RFCs for the three largest patient clusters and also compared contributory biomarkers across the split patient populations. For example, we compared the biomarkers that contributed to the RFC for the 101 smokers to the biomarkers that contributed to the RFC for the 56 non-smokers. Similarly, we compared biomarkers that contributed to RFCs across gender, history of stone disease, history of BPE, anti-hypertensive medication, anti-platelet medication, and anti-ulcer medication.

## Results and discussion

### Non-random distribution of final diagnoses across patient clusters

**Figure 1:**
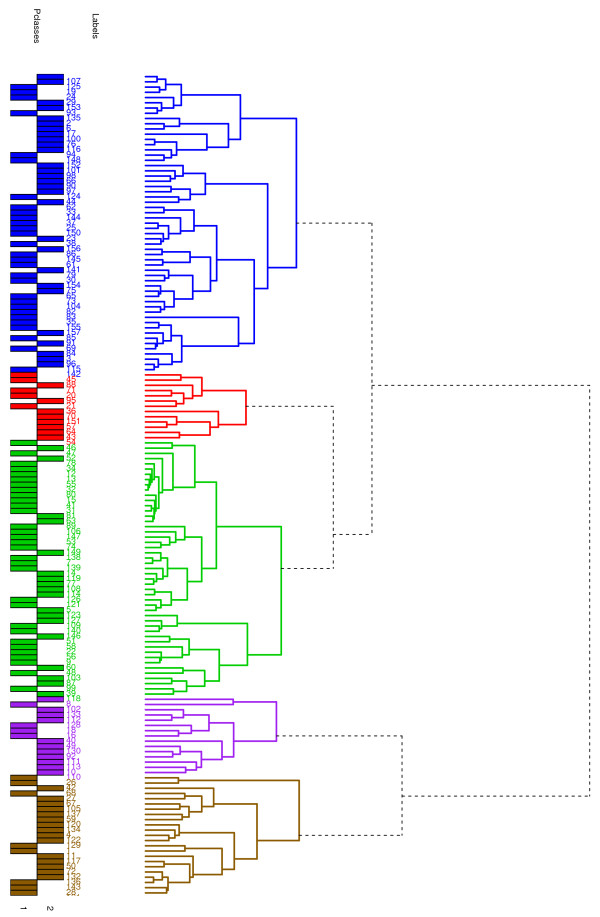
**Hierarchical clustering of the 157 patients based on individual patient biomarker profiles**. Hierarchical clustering of the 157 patients, on the basis of individual patient biomarker profiles, identified five distinct patient clusters as illustrated in this dendrogram. These clusters have (from top to bottom) 57 (28) (blue), 13 (8) (red), 49 (18) (green), 15 (11) (purple) and 23 (15) (gold) patients in each cluster. The number in brackets is the number of patients with urothelial cancer (UC) in the corresponding cluster. UC and control patients were evenly distributed across the five patient clusters. Pclass = 1 corresponds to control patients, that is, hematuric patients who were negative for investigations for UC. Pclass = 2 corresponds to UC patients.

**Figure 2:**
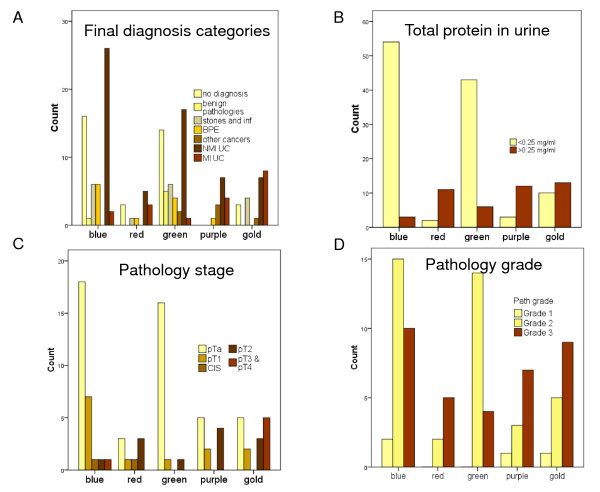
**Cancer-risk characteristics across the patient clusters**. The final diagnosis categories were non-randomly distributed across the five patient clusters identified in Figure 1. The blue and green patient clusters were significantly enriched for patients with 'low cancer-risk' characteristics (bars in yellow) while the red, purple and gold clusters were significantly enriched for patients with 'high cancer-risk' characteristics (bars in dark brown). (**A**) The patient counts, from left to right, within 'no diagnosis', 'benign pathologies', 'stones and inflammation', 'benign prostate enlargement' (BPE), 'other cancers', 'non-muscle invasive urothelial cancer' (NMI UC) and 'muscle invasive urothelial cancer' (MI UC) are illustrated for each of the five patient clusters. Following agglomerative clustering 30/36 (83%) patients within the 'no diagnosis' category were in either the blue or green patient clusters (yellow bars). (**B**) The numbers of patients with normal protein levels are shown by yellow bars. Most patients with normal protein levels fell within the blue (54/112 (48%)) and green clusters (43/112 (38%)). (**C**) The numbers of patients with pTa stage UC are shown by yellow bars. Within the blue and green patient clusters, 18/28 (64%) and 16/18 (89%), respectively, of the patients with UC had pTa disease (yellow bars). In contrast, when the red, purple and gold patient clusters were combined, 16/34 (47%) of the UC patients had high stage disease (dark brown bars). (**D**) The number of patients with Grade 3 UC is shown by dark brown bars. Within the red, purple and gold patient clusters 5/7 (71%), 7/11 (64%), and 9/15 (60%), respectively, had Grade 3 UC. In comparison, 10/27 (37%) and 4/18 (22%), respectively, in the blue and green patient clusters had grade 3 UC (dark brown bars).

When we clustered the 157 patients on the basis of their individual patient biomarker profiles, this resulted in five patient clusters (Figure 1). We observed that the final diagnosis categories were non-randomly distributed across the patient clusters (Figure 2A).

### Non-random distribution of cancer-risk characteristics across patient clusters

Further, we observed that the red, purple and gold patient clusters illustrated in Figure 1, were enriched with patients with 'high cancer-risk' characteristics [2,4,21]. Conversely, the blue and green patient clusters were enriched with patients with 'low cancer-risk' characteristics (Figure 2). On the basis of these observations we designated the red, purple and gold natural patient clusters as 'high-risk' and the blue and green patient clusters as 'low-risk'.

Prior to chi-square analyses we grouped the 'no diagnosis', 'benign pathologies', 'stones and inflammation' and 'BPE' categories together as non-life threatening diagnoses. Similarly, we grouped the cancer patients, that is, 'other cancers', 'NMI UC' and 'MI UC' together as life threatening diagnoses (Table 1). There was a significant difference in life threatening diagnoses between 'low-risk' and 'high-risk' patient clusters (45.3% versus 74.5%, *P *= 0.001). In addition, there were significant differences in proteinuria (8.5% versus 70.6%, *P *<0.001); MI UC (6.5% versus 44.1%, *P *= 0.001); grade 3 UC (31.1% versus 63.6%, *P *= 0.006); and malignant cytology (14.1% versus 48.9%, *P *= 0.001) between 'low-risk' and 'high-risk' patient clusters (Figure 2).

In Figure 2, the yellow bars represent 'low cancer-risk' characteristics, that is, 'no diagnosis', 'benign pathology', '<0.25 mg/ml total urinary protein', 'pTa stage UC' and 'grades 1 and 2 UC'. In contrast, the dark brown bars represent 'high cancer-risk' characteristics, that is, 'other cancers', 'NMI UC', 'MI UC', 'proteinuria', '≥ pT2 stage UC' and 'Grade 3 UC'. There were proportionately more patients in the yellow bars in the blue and green patient clusters in comparison to the proportions recorded within yellow bars within the red, purple and gold patient clusters. In Figure 2A, 30/36 (83%) patients with a final diagnosis of 'no diagnosis' fell within the 'low-risk' patient clusters. In the 'high-risk' patient clusters, 15/51 (29%) patients were in the 'MI UC' final diagnosis category (dark brown bars) (Figure 2A). We speculate that the six patients with a final diagnosis of 'no diagnosis' who clustered into the 'high-risk' patient clusters, could have undetected serious disease, for example, kidney disease [22,23] or another cancer. Unfortunately, we could not explore this possibility because we did not have ethical approval to follow-up the patients. In Figure 2B, 97/106 (92%) patients in the 'low-risk' patient clusters had normal urinary protein levels (yellow bars). In contrast, in the 'high-risk' patient clusters, 36/51 (71%) patients had proteinuria (dark brown bars) (Figure 2B). Ideally, hematuric patients with significant proteinuria should be referred to nephrology [21] to be investigated for kidney disease [21-23]. In Figure 2C, pathological stages are represented by bars from left to right, that is, starting with pTa (yellow bars) and progressing through to dark brown bars (pT3/pT4 stage UC). Although 28 patients within the 'low-risk' blue cluster and 18 patients in the 'low-risk' green cluster had UC, 18/28 (64%) and 16/18 (89%) of these UC, respectively, were stage pTa (yellow bars) (Figure 2C). Further, 15/18 (73%) pTa tumors in the blue cluster and 14/16 (88%) pTa tumors in the green cluster were ≤ pTaG2, that is, very low risk tumors [2]. Forty-four percent, that is, 15/34 of the UC patients in the red, purple and gold clusters had tumors ≥ pT2, which would be deemed high-risk [4] (brown bars) (Figure 2C). As we have previously discussed, there is molecular heterogeneity within the same tumor stage and it is possible that some of the pT1 and CIS tumors falling within the red, purple and gold clusters could have predisposing molecular profiles for progression. Further, it is important to emphasize that the division of UC tumors into NMI and MI is arbitrary and perhaps too simplistic. For example, there will be a significant difference in risk between a pT1 tumor with minimal submucosal invasion and a pT1 tumor with extensive submucosal invasion with the concomitant risk of lymphovascular invasion. Grade reflects the degree of differentiation within a tumor. When we explored the pathological grades of the UC tumors, 21/33 (64%) UC patients in the 'high-risk' patient clusters had grade 3 disease (dark brown bars) compared to 14/45 (31%) in the 'low-risk' clusters (Figure 2D). In addition, we found that there were significant differences in malignant cytology (14.1% versus 48.9%, *P *= 0.001) between 'low-risk' and 'high-risk' patient clusters.

### Reduction of the complexity of the biomarker data

**Figure 3:**
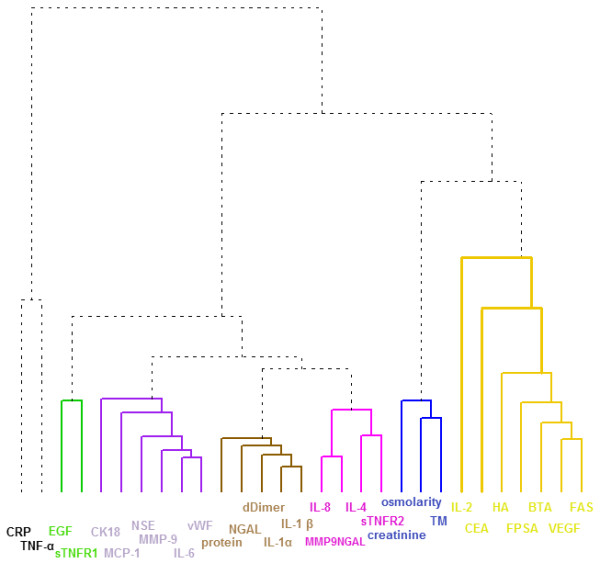
**Hierarchial clustering of the 29 biomarkers**. This dendrogram illustrates seven distinct biomarker clusters containing (from left to right): 2 (black), 2 (green), 6 (purple), 5 (gold), 4 (pink), 3 (blue) and 7 (yellow) biomarkers each. Two of the biomarker clusters comprised predominantly inflammatory proteins. For example, the brown cluster comprised D-dimer, interleukin-1α (IL-1α), interleukin-1β (IL-1β), neutrophil-associated gelatinase lipocalin (NGAL) and total protein. BTA, bladder tumor antigen; CEA, carcino-embryonic antigen; CK18, cytokeratin 18; CRP, C-reactive protein; EGF, epidermal growth factor; FPSA, free prostate specific antigen; HA, hyaluronidase; MCP-1, monocyte chemoattractant protein-1; MMP-9, matrix metalloproteinase 9; NSE, neuron specific enolase; sTNFR1, soluble TNF receptor 1; TM, thrombomodulin; TNFα, tumor necrosis factor α; VEGF, vascular endothelial growth factor; vWF, von Willeband factor.

We used hierarchical clustering to identify the most informative set of biomarkers for use as feature vectors for UC diagnostic classifiers. Hierarchical clustering identified seven biomarker clusters consisting of *N_b _*= (2, 2, 6, 5, 4, 3, 7) biomarkers (Figure 3). We assumed that biomarkers within individual clusters would contain redundant biological information about the patients and that it was sufficient to select one biomarker to represent each cluster. Overall, this provided us with a systematic way to estimate the number of representative biomarkers, which could be considered as the effective dimension of the biomarker-space. From this it follows that the total number of combinations is only 10,080 as given by

$$N_{C}=\prod_{i=1}^{7}N_{b}(i)=10080$$

each corresponding to a 7-tuple of biomarkers. Hence, the grouping of biomarkers into seven groups broke down the combinatorial complexity of the overall problem, allowing us to conduct an exhaustive search in this constraint set of biomarkers. In contrast, an unconstrained, exhaustive search would not have been feasible because the number of unconstrained feature combinations for up-to 7-dimensional feature vectors is larger than 2.1 million, as given by

$$N_{T}(29)=\sum_{k=1}^{29}\left(\begin{array}{c} 29\\ k \end{array}\right)$$

$\left(\begin{array}{c} 29\\ k \end{array}\right)$ is the binomial coefficient.

This is more than two orders of magnitude larger than N*_C _*making an exhaustive search computationally infeasible.

For all possible *N_C _*= 10,080 biomarker combinations, we determined the classification error and the AUROC of RFCs for each of the following: (1) all the 157 patients, (2) the three largest patient clusters from Figure 1, and (3) 14 subpopulations which were split on the basis of clinical or demographic parameters.

### Contributory biomarkers to UC diagnostic classifiers for the low-risk patient clusters were similar

Only two of the patient clusters, those shown in blue and green in Figure 1, contained sufficient numbers, that is, 57 and 48, to train a RFC. However, for reasons of comparison, we also trained a RFC for the gold cluster, which contained 23 patients, 15 of whom were diagnosed with UC (Figure 2). We found that 4/7 biomarkers were the same in the diagnostic classifiers for the blue and green patient clusters suggesting that these patient clusters had biological similarities. This is interesting because we had designated patients within both of these clusters as 'low-risk'. Further, only 2/7 and 1/7 of the biomarkers, which contributed to the blue and green low-risk clusters, respectively, also contributed to the classifier for the gold cluster. This would suggest that the gold patient cluster had significantly different underlying biological properties in comparison to the blue and green clusters. These observations would concur with our risk stratification hypothesis. The standard deviation of the classification error and of the AUROC for this smaller gold cluster, in comparison to the blue and green patient clusters, increased by approximately 30 % (Table 3).

**Table 3:** Random Forest Classifiers for patient clusters and clinical subpopulations.

Variable description	Sub populations	Biomarkers	Classification error (SD)	AUROC (SD)
All 157 hematuria patients	controls n = 77 UC n = 80	CRP, EGF, IL-6, IL-1α, MMP9NGAL, osmolarity, CEA	0.203 (0.017)	0.766 (0.152)

Patient clusters^a^	blue n = 57 (28)	TNFα, EGF, NSE, NGAL, MMP9NGAL, TM, FAS	0.155 (0.029)	0.800 (0.258)
	
	green n = 49 (18)	TNFα, EGF, IL-6, IL-1α, MMP9NGAL, TM, CEA	0.204 (0.037)	0.825 (0.264)
	
	gold n = 23 (15)	CRP, sTNFR1, vWF, IL-1α, MMP9NGAL, creatinine, BTA	0.245 (0.049)	0.700 (0.349)

Clinical subpopulations				

Smoking	smokers n = 101 (60)	CRP, EGF, MMP9, IL-1α, IL-4, TM, IL-2	0.276 (0.027)	0.770 (0.117)
	
	non- smokers n = 56 (20)	TNFα, sTNFR1, IL-6, IL-1α, MMP9NGAL, creatinine, CEA	0.156 (0.027)	0.783 (0.159)

Gender	males n = 120 (65)	CRP, EGF, CK18, IL-1β, IL-8, creatinine, IL-2	0.272 (0.030)	0.753 (0.117)
	
	females n = 37 (15)	CRP, EGF, IL-6, dDimer, MMP9NGAL, osmolarity, CEA	0.181 (0.054)	0.830 (0.146)

Hx stone disease	yes n = 30 (14)	CRP, sTNFR1, CK18, IL-1α, IL-8, creatinine, VEGF	0.322 (0.062)	0.738 (0.194)
	
	no n = 127 (66)	CRP, EGF, IL-6, IL-1α, MMP9NGAL, creatinine, CEA	0.186 (0.015)	0.817 (0.117)

Hx BPE	yes n = 30 (14)	CRP, EGF, IL-6, IL-1α, MMP9NGAL, TM, CEA	0.192 (0.018)	0.826 (0.148)
	
	no n = 127 (66)	CRP, EGF, CK18, NGAL, MMP9NGAL, creatinine, BTA	0.266 (0.061)	0.788 (0.169)

Anti-hypertensive medication	on medication n = 73 (51)	TNFα, EGF, IL-6, protein, MMP9NGAL, creatinine, CEA	0.211 (0.025)	0.731 (0.161)
	
	no medication n = 83 (28)	TNFα, sTNFR1, IL-6, NGAL, IL-8, TM, CEA	0.145 (0.028)	0.810 (0.132)

Anti-platelet medication	on medication n = 37 (25)	TNFα, EGF, IL-6, protein, IL-8, osmolarity, CEA	0.215 (0.019)	0.780 (0.141)
	
	no medication n = 118 (53)	CRP, EGF, MCP-1, protein, MMP9NGAL, TM, FPSA	0.160 (0.046)	0.843 (0.153)

Anti-ulcer medication	on medication n = 33 (17)	CRP, EGF, IL-6, IL-1α, IL-8, TM, CEA	0.220 (0.018)	0.827 (0.118)
	
	no medication n = 123 (62)	CRP, EGF, vWF, IL-1β, MMP9NGAL, TM, HA	0.259 (0.072)	0.812 (0.168)

Using the clusters of biomarkers as a feature set, we determined the classification error and the area under the receiver operating characteristic curve (AUROC) of urothelial cancer (UC) diagnostic classifiers for all possible biomarker combinations for all 157 hematuria patients; for 3/5 of the patient clusters; and for 14 subpopulations split on the basis of smoking, gender, history of stone disease, history of benign prostate enlargement (BPE), or anti-hypertensive, anti-platelet or anti-ulcer medications. Therefore, one biomarker from each of the seven clusters illustrated in the biomarker dendrogram (Figure 3), was represented in each classifier. The classification errors in the clinically split populations were very similar to those obtained for the patient clusters. ^a^Only two of the natural patient subpopulations, those shown in blue and green in Figure 1, contained sufficient numbers to train a Random Forest Classifier (RFC). For reasons of comparison, we also trained a RFC for the gold cluster. Four of the seven biomarkers were the same in the diagnostic classifiers for the blue and green patient clusters suggesting biological similarities. The numbers in brackets in the second column indicate the number of patients with UC. BTA, bladder tumor antigen; CEA, carcino-embryonic antigen; CK18, cytokeratin 18; CRP, C-reactive protein; EGF, epidermal growth factor; FPSA, free prostate specific antigen; IL, interleukin; HA, hyaluronidase; MMP-9, matrix metalloproteinase 9; NGAL, neutrophil-associated gelatinase lipocalin; NSE, neuron specific enolase; SD, standard deviation; sTNFR1, soluble tumor necrosis factor receptor 1; TM, thrombomodulin; TNFα, tumor necrosis factor α; VEGF, vascular endothelial growth factor; vWF, Von Willeband factor.

### Contributory biomarkers to UC diagnostic classifiers across clinically split patient subpopulations were different

When we determined classification errors and AUROCs of UC diagnostic RFCs for 14 clinically defined subpopulations we observed the highest AUROC = 0.843 (averaged over 100 repetitions) in the classifier for patients not taking anti-platelet medication (n = 118). For the clinically split subpopulations, we found that when specific biomarkers contributed to the UC diagnostic RFC for one clinically relevant subpopulation, they were less likely to contribute to the RFC for the complementary subpopulation. For example, compare the biomarkers across patient subpopulations taking anti-platelet medication to those not on the medication (Table 3).

### Biomarkers associated with inflammatory conditions predominated two of the biomarker clusters

Biomarkers associated with inflammatory conditions predominated the black and brown biomarker clusters (Figure 3). The black cluster contained C-reactive protein (CRP) and TNFα. The brown cluster comprised D-dimer, interleukin-1α, interleukin-1β, neutrophil-associated gelatinase lipocalin (NGAL) and total urinary protein. The latter five biomarkers were significantly elevated in urine from patients in the 'high-risk' patient clusters (Mann Whitney U, *P *<0.001) (Table 4). NGAL is expressed by neutrophils and its main biological function is inhibition of bacterial growth [24]. NGAL, being resistant to degradation, is readily excreted in urine, both in its free form and in complex with MMP-9, which may protect it from degradation [24]. NGAL is also a useful biomarker of acute kidney disease [23]. Since the prevalence of kidney disease is one in six adults [25], NGAL should perhaps be an important consideration in urinary biomarker studies on patient populations which include high proportions of patients >50 years old. In our analyses, significantly higher NGAL levels were recorded in the purple patient subpopulation (1,379 ng/ml), 14/15 of whom had cancer, compared to levels measured in the patients in the gold group (464 ng/ml) (Table 4) who had a greater diversity of final diagnoses (Figure 2A) (Mann Whitney U; *P *= 0.012).

**Table 4:** Median biomarker levels in patient clusters.

Biomarker (units)	Median (inter quartile range) in patient clusters				
	
	blue	red	green	purple	gold
BLACK CLUSTER					

CRP (ng/ml)	1.05 (0.74 to 1.33)	0.86 (<0.67 to 0.91)	1.06 (0.84 to 1.25)	<0.67 (<0.67 to 0.83)	0.75 (<0.67 to 0.90)
TNFα (pg/ml)	10.52 (7.78 to 13.25)	9.07 (7.36 to 9.79)	9.54 (7.46 to 11.78)	11.66 (8.95 to 15.48)	10.20 (8.31 to 12.820)

GREEN CLUSTER					

EGF (pg/ml)	7,056 (4,965 to 13,752)	3,633 (1,874 to 5,992)	6,477 (2,784 to 10,943)	3,722.33 (3,058 to 4,956)	13,826 ( 9,488 to 20,332)
sTNFR1(pg/ml)	0.67 (0.41 to 1.04)	0.75 (0.47 to 1.05)	0.57 (0.24 to 1.61)	0.74 (0.45 to 1.06)	1.60 (0.97 to 2.54)

PURPLE CLUSTER					

CK18 (ng/ml)	2.30 (0.71 to 3.59)	1.22 (0.42 to 2.78)	2.03 (0.75 to 4.33)	8.97 (2.88 to 21.43)	6.43 (2.67 to 10.28)
MCP-1 (pg/ml)	112 (38 to 212)	73 (41 to 141)	67 (28 to 113)	269 (118 to 871)	237 (106 to 550)
NSE (ng/ml)	IQR below LOD	0.28 (<0.26 to 0.92)	IQR below LOD	1.72 (<0.26 to 18.32)	0.51 (< 0.26 to 2.37)
MMP-9 (ng/ml)	IQR below LOD	IQR below LOD	IQR below LOD	15.15 (6.57 to 50.81)	IQR below LOD
IL-6 (pg/ml)	1.37 (<1.20 to 3.60)	12.93 (3.27 to 26.67)	<1.20(<1.20 to 2.50)	194.33 (16.43 to 577.33)	40.80 (4.80 to 196.67)
vWF (IU/ml) purple	0.01 (0.01 to 0.02)	0.01 (0.00 to 0.01)	0.01 (0.01 to 0.01)	0.01 (0.01 to 0.02)	0.01 (0.01 to 0.03)

GOLD CLUSTER					

Protein (mg/ml)	0.07 (0.05 to 0.11)	0.44(0.29 to 0.60)	0.08 (0.05 to 0.12)	0.59 (0.25 to 0.93)	0.30 (0.09 to 1.00)
NGAL (ng/ml)	123 (92 to 212)	192 (146 to 297)	110 (74 to 148)	1,379 (602 to 1922)	464 (108 to 1368)
D-dimer (ng/ml)	<2.10 (<2.10 to 5.02)	47.01 (11.80 to 138.27)	<2.10 (<2.10 to 3.62)	597.89 (62.16 to 1493.69)	58.35 (<2.10 to 559.38)
IL-1α (pg/ml)	0.90 (0.90 to 2.52)	2.42 (1.01 to 3.53)	0.90 (0.90 to 1.01)	21.35 (5.80 to 30.93)	2.47 (0.90 to 81.00)
IL-1β (pg/ml)	IQR below LOD	IQR below LOD	IQR below LOD	17.80 (5.46 to 78.87)	<1.60 (<1.60 to 19.12)

PINK CLUSTER					

IL-8 (pg/ml)	32.40 (7.93 to 265.83)	292.67 (117.33 to 604.33)	28.63 (7.90 to 135.33)	2,900 (2,064 to 2,900)	875.67 (48.40 to 2,900)
MMP9NGAL	0.09 (0.08 to 0.10)	0.16 (0.10 to 0.29)	0.07 (0.07 to 0.09)	0.29 (0.23 to 0.48)	0.23 (0.09 to 0.29)
IL-4 (pg/ml)	IQR below LOD	IQR below LOD	IQR below LOD	<6.60 (<6.60 to 6.80)	IQR below LOD
sTNFR2 (pg/ml)	IQR below LOD	<0.15 (<0.15 to 0.26)	<0.15 (<0.15 to 0.26)	IQR below LOD	<0.15 (<0.15 to 0.61)

BLUE CLUSTER					

creatinine (µmol/L)	9,608 (7,961 to 13,360)	5,605 (4,454 to 11,945)	7,115 (3,868 (12,595)	7,600 (5,360 to 8,625)	14,087 (12,405 to 17,245)
osmolarity (mOsm)	536 (450 to 741)	462 (276 to 560)	526 (278 to 675)	404 (314 to 482)	644 (567 to 7,840)
TM (ng/ml)blue	4.08 (3.19 to 4.97)	3.97 (1.55 to 5.69)	4.00 (1.74 to 5.68)	3.49 (2.65 to 4.00)	6.30 (5.34 to 8.86)

YELLOW CLUSTER					

IL-2 (pg/ml)	5.61 (5.21 to 5.92)	5.24 (5.02 to 5.65)	5.45 (5.20 to 6.27)	6.89 (5.99 to 7.24)	5.99 (5.65 to 7.20)
CEA (ng/ml)	1.57 (1.16 to 2.58)	1.59 (1.15 to 3.23)	1.36 (0.87 to 2.10)	1.77 (1.30 to 2.39)	1.37 (0.89 to 2.80)
HA (ng/ml)	685 (439 to 866)	835 (595 to 1005)	594 (282 to 900)	1,569 (1,143 to 1,846)	1,258 (883 to 1,712)
FPSA (ng/ml)	0.09 (0.04 to 0.21)	0.05 (0.04 to 0.23)	0.07 (0.04 to 0.12)	0.13 (0.07 to 0.30)	0.05 (0.04 to 0.10)
BTA (U/ml)	8.52 (2.57 to 38.96)	248.01 (206.82 to 394.15)	6.27 (1.21 to 17.92)	278.41 (226.40 to 504.33)	213.00 (15.24 to 476 .28)
VEGF (ng/ml)	88 (37 to 271)	96 (76 to 220)	78 (38 to 122)	1,266 (414 to 1,500)	253 (79 to 621)
FAS (pg/ml)	64 (42 to 96)	83 (60 to 128)	56 (37 to 86)	214 (106 to 475)	200 (96 to 279)

The median level and the inter-quartile range (IQR) of each biomarker in each patient cluster are shown. The biomarkers are grouped vertically to reflect how they appear in the biomarker cluster dendrogram (Figure 3). BTA, bladder tumor antigen; CEA, carcino-embryonic antigen; CK18, cytokeratin 18; CRP, C-reactive protein; EGF, epidermal growth factor; FPSA, free prostate specific antigen; HA, hyaluronidase; IL, interleukin; LOD, limit of detection; MCP-1, monocyte chemoattractant protein-1; MMP-9, matrix metalloproteinase 9; NGAL, neutrophil-associated gelatinase lipocalin; NSE, neuron specific enolase; sTNFR1, soluble tumor necrosis factor receptor 1; sTNFR2, soluble tumor necrosis factor receptor 2; TM, thrombomodulin; TNFα, tumor necrosis factor α; VEGF, vascular endothelial growth factor; vWF, Von Willeband factor.

Median EGF levels were significantly higher in the gold patient cluster (14 µg/ml) in comparison to the purple patient cluster (4 µg/ml) (Mann Whitney U; *P *<0.001) (Table 4). Interestingly, 9/23 patients in the gold patient cluster had ≥ pT1G3 UC and the purple patient cluster included cancers other than UC (Figure 2). Bladder cancer risk and survival have been associated with genetic variation in the Epidermal growth factor receptor (EGFR) pathway [26].

### Translation of risk and diagnostic classifiers from systems biology to the clinic

We have described how hierarchical clustering, conducted on the basis of individual patient biomarker profiles, identified patient clusters and how cancer-associated risk characteristics were non-randomly distributed across these clusters (Figures 1 and 2 and Tables 5, 6, 7, 8, 9, 10). These findings suggest that it should be possible to define risk classifiers which could be informative at the point of triage of hematuric patients. This approach could have the potential to significantly improve healthcare outcomes for patients with hematuria.

**Table 5:** Final diagnoses across the patient clusters.

patient clusters	final diagnosis categories							total
		
	no diagnosis	benign pathologies	stones and/orinflammation	BPE	other cancers	NMI UC	MI UC	
blue	16	1	6	6	0	26	2	57
red	3	0	1	1	0	5	3	13
green	14	5	6	4	2	17	1	49
purple	0	0	0	1	3	7	4	15
gold	3	0	4	0	1	7	8	23

BPE, benign prostate enlargement; MI UC, muscle invasive urothelial cancer; NMI UC, non-muscle invasive urothelial cancer.

**Table 6:** Total urinary protein across the patient clusters.

patient clusters	total urinary protein (mg/ml)		total
		
	<0.25	>0.25	
blue	54	3	57
red	2	11	13
green	43	6	49
purple	3	12	15
gold	10	13	23

**Table 7:** Pathology stages of the urothelial carcinomas across the patient clusters.

patient clusters	pathology stage								total
		
	pTa	pT1	pT2a	pT2b	pT3a	pT3b	pT4a	CIS	
blue	18	7	1	0	0	0	1	1	28
red	3	1	3	0	0	0	0	1	8
green	16	1	1	0	0	0	0	0	18
purple	5	2	2	2	0	0	0	0	11
gold	5	2	3	0	1	3	1	0	15

CIS, carcinoma *in situ*.

**Table 8:** Pathology grades of the urothelial carcinomas across the patient clusters.

patient clusters	pathology grade			total
		
	grade 1	grade 2	grade 3	
blue	2	15	10	27
red	0	2	5	7
green	0	14	4	18
purple	1	3	7	11
gold	1	5	9	15

**Table 9:** Cytology diagnosis across the patient clusters.

patient clusters	cytology		total
		
	no evidence of malignancy	malignant	
blue	38	11	49
red	7	6	13
green	41	2	43
purple	8	6	14
gold	9	11	20

**Table 10:** Pathological grades of the Ta stage urothelial carcinomas across the patient clusters.

patient clusters	pathology grades in pTa tumors			total
		
	grade 1	grade 2	grade 3	
blue	2	13	3	18
red	0	2	1	3
green	0	14	2	16
purple	1	3	1	5
gold	1	3	1	5

**Figure 4:**
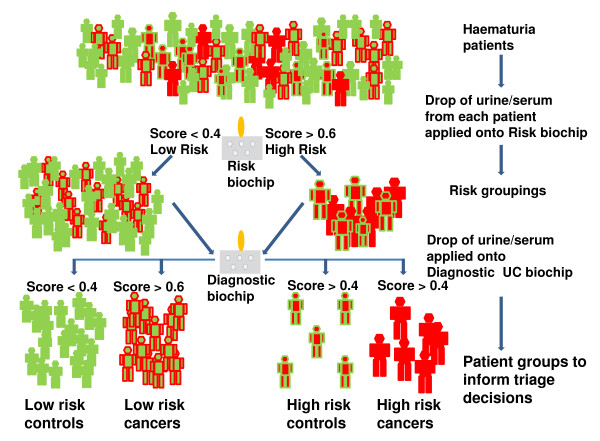
**Translation of classifiers into biochip format for risk stratification of hematuria patients**. In the future when a patient with hematuria presents in primary care, their urine and serum samples could be sent for evaluation using biochips (grey oblongs). One biochip could be created for risk stratification and one biochip for the diagnosis of UC. Each biochip would be formatted with approximately six antibody spots, referred to as test regions. The underlying concept of these biochips is based on procedures similar to an ELISA, that is, light readings are generated from each test region which are proportional to the bound protein that is present in each patient's sample. Computer software would generate a score between 0 and 1 for each patient's sample. For the risk biochip, scores <0.4 would suggest a low risk of serious disease, while scores >0.6 would suggest a high risk of serious disease. The patient could then be designated low-risk (green) or high-risk (red) risk. Patients would then be screened using a second biochip, this time a UC diagnostic biochip. Similarly, scores <0.4 from the UC diagnostic biochip would suggest that it was unlikely that the patient would have bladder cancer while scores >0.6 would suggest that the patient requires further investigations to check for the presence of UC. The scores from both biochips would be interpreted alongside clinical parameters. The patient's clinician would then make a triage decision for that patient which would be informed by the biochip scores. For example, a high-risk UC patient (all red) could obtain a score >0.6 on the scale ranging from 0 to 1 for both biochips and likewise a low-risk control could receive a score <0.4 for both biochips. ELISA, enzyme-linked immunosorbent assay; UC, urothelial cancer.

Biochip array technology [17] allows rapid and simultaneous measurement of the levels of multiple biomarkers. This technology will facilitate the translation of protein-based classifiers, as described in this manuscript, from the laboratory to the clinic [27]. Antibodies, raised against biomarkers contributing to an individual classifier, can be formatted onto a single biochip. We predict that risk stratification biochips and UC diagnostic biochips could be created and validated in the near future [28]. In clinical practice, scores between 0 and 1, from the risk and diagnostic UC biochips would make it possible to designate each patient with hematuria as a 'low-risk control', a 'high-risk control', a 'low-risk UC' or a 'high-risk UC (Figure 4). Scores <0.4 obtained using the risk biochip would suggest that the likelihood of serious disease was low. Similarly, a score <0.4 obtained using the UC diagnostic biochip would suggest that it was unlikely that the patient had UC. In contrast, scores >0.6 from the risk or diagnostic biochip would be suggestive of serious disease or UC, respectively. Scores between 0.4 and 0.6 could be interpreted as indicative of potential risk and the possibility of UC.

If specificities and sensitivities for both biochips were >90%, this would mean a high-risk cancer patient would have a 1:10 chance of being wrongly classified as low-risk and subsequently a 1:10 chance of being wrongly classified as a control. In this scenario, out of 1,000 high-risk cancer patients approximately 810 would be correctly classified as high-risk cancers, approximately 90 as high-risk controls, approximately 90 as low-risk cancers and approximately 10 as low-risk controls (Figure 4). Following biochip analyses, patients with scores ≤0.2 from both biochips and no clinical risk factors, that is, low-risk controls, could be monitored in primary care. This would lead to a reduction in the number of cystoscopies in these patients. In another scenario, a proportion of patients might be assigned as high-risk control patients following analyses of their samples using the biochips. These patients should be investigated further because they could have other diseases, for example, kidney disease which could then be managed appropriately [21]. In this way, improved triage would result in expeditious diagnosis for a greater proportion of patients with hematuria who would then receive earlier and more effective therapeutic interventions. This would represent a significant healthcare improvement [29].

Single biomarkers have failed to be diagnostic for hematuria and many other complex diseases. Panels of biomarkers, in addition to clinical information, provide a large array of patient data that can be highly informative and have potential for diagnostic and prognostic decision making. However, the difficulties to date with large amounts of patient biomarker data are that they do not manage or group all patients in a clinically meaningful way. Systems biology is a developing technology [30] that has evolved new and different ways to analyze very large and complex datasets, such as those relating to sequencing of the genome and those collected from complex diseases. We have described how patients with hematuria naturally cluster into risk groupings on the basis of their individual biomarker profiles. This challenges the current practice in hematuria clinics which prioritizes diagnosis of patients with bladder cancer. Patients in the 'high-risk' clusters included controls, that is, patients without bladder cancer. However these 'controls' may have other cancers or may have neoplasms at very early stages of carcinogenesis, that is, below the size threshold for detection. Because cystoscopy is not a perfect diagnostic tool and because there is an urgent need to identify all patients with serious disease at the hematuria clinic, the findings in this paper represent a significant advance in the approach to triage and diagnosis of hematuria patients.

## Conclusions

When we clustered patients with hematuria on the basis of their individual patient biomarker profiles, we identified five patient clusters. We observed that the final diagnoses for the 157 patients with hematuria were non-randomly distributed across these patient clusters. Other 'high cancer-risk' characteristics, that is, proteinuria, pathological stage, pathological grade and malignant cytology were also non-randomly distributed across the patient clusters. Indeed, we identified three patient clusters that were enriched with patients who harbored 'high cancer-risk' characteristics and two patient clusters that were enriched with patients with 'low cancer-risk' characteristics. These findings indicate the feasibility of creating risk classifiers that could inform the triage of patients with hematuria. Risk classifiers could improve decision-making at the point of triage. This would result in a more accurate and timely diagnosis for patients with serious disease thus improving outcomes for a greater proportion of patients [1,2,29].

## Abbreviations

AUROC: area under the receiver operating characteristic curve; BPE: benign prostate enlargement; BTA: bladder tumor antigen; CEA: carcinoembryonic antigen; CIS: carcinoma *in situ; *CK18: cytokeratin 18; CRP: C-reactive protein; EGF: epidermal growth factor; ELISA: enzyme-linked immunosorbent assay; FDA: Food and Drug Administration; FPSA: free prostate specific antigen; HA: hyaluronidase; IL: interleukin; IQR: inter-quartile range; LOD: limit of detection; MCP-1: monocyte chemoattractant protein-1; MI: muscle invasive; MMP-9: matrix metalloproteinase-9; NGAL: neutrophil-associated gelatinase lipocalin; NMI: non-muscle invasive; NMP22: nuclear matrix protein 22; NSE: neuron specific enolase; PSA: prostate specific antigen; RCC: renal cell carcinoma; RFC: Random Forest Classifiers; SD: standard deviation; STARD: Standards for Reporting of Diagnostic Accuracy; TCC: transitional cell carcinoma; TNFα: tumor necrosis factor alpha; sTNFR: soluble TNFα receptor; TM: thrombomodulin; UC: urothelial cancer; UTI: urinary tract infection; VEGF: vascular endothelial growth factor; vWF: von Willebrand factor.

## Competing interests

We declare that MWR and CNR are employees of Randox Laboratories Ltd who undertook the biomarker analyses using Biochip Array Technology. Randox Laboratories funded the salary of FA who recruited the patients to the case control study over two years. MWR, CNR, and KEW are named inventors on British Patent No 0916193.6, which protects the biomarkers in algorithms previously published in Cancer 2012 DOI: 10.1002/cncr.26544. FES, RdeMS, BD, OR, LW, HFOK, DOR, NHA and TN declare that they have no competing interests.

## Authors' contributions

FES performed statistical analyses, developed concepts, interpreted the statistical analyses and drafted the manuscript. FA was involved in the conception and design of the case control study and read drafts of the manuscript. RdeMS performed components of the statistical analyses and interpreted the same. BD interpreted the findings, wrote components of the manuscript, read drafts of the manuscript and contributed to discussions that shaped the manuscript. MR performed the protein analyses, read drafts of the manuscript and contributed to discussions that shaped the manuscript. CR performed the protein analyses, read drafts of the manuscript and contributed to discussions that shaped the manuscript. OR participated in the statistical analyses of the data and wrote sections of the manuscript. LW participated in the statistical analyses of the data, wrote sections of the manuscript and read drafts of the manuscript. HFOK was involved in the conception and design of the case control study and read drafts of the manuscript. DOR assessed the tumor pathology and read drafts of the manuscript. NHA assessed the cytology and was involved in the conception and design of the study. TN read drafts of the manuscript and contributed to discussions about the clinical significance. KW was involved in the conception and designed the case control study, undertook and interpreted the statistical analyses, developed and tested novel concepts, and contributed to discussions that shaped the manuscript and wrote final drafts of the manuscript. All authors read and approved the final manuscript.

## Pre-publication history

The pre-publication history for this paper can be accessed here:

http://www.biomedcentral.com/1741-7015/11/12/prepub
